# MUC1 Expression by Immunohistochemistry Is Associated with Adverse Pathologic Features in Prostate Cancer: A Multi-Institutional Study

**DOI:** 10.1371/journal.pone.0165236

**Published:** 2016-11-15

**Authors:** Okyaz Eminaga, Wei Wei, Sarah J. Hawley, Heidi Auman, Lisa F. Newcomb, Jeff Simko, Antonio Hurtado-Coll, Dean A. Troyer, Peter R. Carroll, Martin E. Gleave, Daniel W. Lin, Peter S. Nelson, Ian M. Thompson, Lawrence D. True, Jesse K. McKenney, Ziding Feng, Ladan Fazli, James D. Brooks

**Affiliations:** 1 Department of Urology, Stanford University, Stanford, CA, United States of America; 2 Department of Urology, University Hospital of Cologne, Cologne, NRW, Germany; 3 The Department of Biostatistics, the University of Texas MD Anderson Cancer Center, Houston, TX, United States of America; 4 Canary Foundation, Canary Center at Stanford, 3155 Porter Drive, Palo Alto, CA, United States of America; 5 Department of Urology, University of Washington Medical Center, Seattle, WA, United States of America; 6 Department of Pathology, University of California San Francisco, San Francisco, CA, United States of America; 7 Department of Urologic Sciences and Vancouver Prostate Centre, Vancouver, BC, Canada; 8 Department of Pathology, University of Texas Health Science Center at San Antonio, San Antonio, TX, United States of America; 9 Eastern Virginia Medical School, Pathology and Microbiology and Molecular Biology, Norfolk, VA, United States of America; 10 Department of Urology, University of California San Francisco, San Francisco, CA, United States of America; 11 Division of Human Biology, Fred Hutchinson Cancer Research Center, Seattle, WA, United States of America; 12 Department of Urology, University of Texas Health Science Center at San Antonio, San Antonio, TX, United States of America; 13 Department of Pathology, University of Washington Medical Center, Seattle, WA, United States of America; 14 Department of Pathology, Cleveland Clinic, Cleveland, Ohio, United States of America; University of Kentucky College of Medicine, UNITED STATES

## Abstract

**Background:**

The uncertainties inherent in clinical measures of prostate cancer (CaP) aggressiveness endorse the investigation of clinically validated tissue biomarkers. MUC1 expression has been previously reported to independently predict aggressive localized prostate cancer. We used a large cohort to validate whether MUC1 protein levels measured by immunohistochemistry (IHC) predict aggressive cancer, recurrence and survival outcomes after radical prostatectomy independent of clinical and pathological parameters.

**Material and Methods:**

MUC1 IHC was performed on a multi-institutional tissue microarray (TMA) resource including 1,326 men with a median follow-up of 5 years. Associations with clinical and pathological parameters were tested by the Chi-square test and the Wilcoxon rank sum test. Relationships with outcome were assessed with univariable and multivariable Cox proportional hazard models and the Log-rank test.

**Results:**

The presence of MUC1 expression was significantly associated with extracapsular extension and higher Gleason score, but not with seminal vesicle invasion, age, positive surgical margins or pre-operative serum PSA levels. In univariable analyses, positive MUC1 staining was significantly associated with a worse recurrence free survival (RFS) (HR: 1.24, CI 1.03–1.49, P = 0.02), although not with disease specific survival (DSS, P>0.5). On multivariable analyses, the presence of positive surgical margins, extracapsular extension, seminal vesicle invasion, as well as higher pre-operative PSA and increasing Gleason score were independently associated with RFS, while MUC1 expression was not. Positive MUC1 expression was not independently associated with disease specific survival (DSS), but was weakly associated with overall survival (OS).

**Conclusion:**

In our large, rigorously designed validation cohort, MUC1 protein expression was associated with adverse pathological features, although it was not an independent predictor of outcome after radical prostatectomy.

## Introduction

Prostate cancer (CaP) is the most frequently diagnosed cancer and the third leading cause of death from cancer among men worldwide [1]. Prostate specific antigen (PSA) testing has been used for screening and disease monitoring, such as in active surveillance or after therapy for CaP. However, for men with clinically localized CaP, PSA cannot reliably predict clinical outcomes, particularly since many men have a PSA level < 10 ng/ml at the time of diagnosis where PSA is not prognostic [2]. Therefore, additional biomarkers that are associated with clinical outcome are needed. The mucin family encompasses a diverse set of high molecular weight glycoproteins characterized by the presence of O-linked oligosaccharides to serine or threonine residues [3, 4]. MUC1 protein expression has been found to be significantly elevated in several cancers including CaP [4, 5] and is usually accompanied by altered glycosylation [6, 7]. In addition, MUC1 expression in cancer is usually characterized by a diffuse cytoplasmic staining pattern compared to apically restricted expression typically found in normal tissues [8–11]. MUC1 over-expression has been reported to allow malignant cells to evade host immunological defenses and to promote metastasis through a loss of cell–cell and cell–extracellular matrix contact [7, 12–16].

In CaP, MUC1 over-expression has been associated with increased risk of recurrence and adverse pathological findings in patients undergoing radical prostatectomy [5, 17–19]. We have developed a multi-institutional Tissue Microarray Resource of radical prostatectomy samples for definitive validation of biomarkers of prognosis that are independent of clinical and pathological features [20]. We have used this resource to validate several tissue-based candidate biomarkers of prognosis and evaluated whether their ability to prognosticate is independent of clinical and pathological features [21–26]. Our goal is to validate candidate biomarkers of prognosis to aid in the identification of patients with increased risk for tumor progression and poor survival outcomes after radical prostatectomy. Based on strong preliminary data implicating MUC1 expression as a marker of adverse outcome in CaP, we evaluated whether MUC1 expression by immunohistochemistry was associated with recurrence and survival after radical prostatectomy.

## Materials and Methods

The study was conducted in accordance with IRB-approved protocols at each participating site (Stanford University, University of California San Francisco, University of Washington, University of British Colombia, University of Texas Health Sciences Center at San Antonio, Eastern Virginia Medical Center) and a materials transfer agreement for sharing of tissue microarrays, clinical information and tissue samples.

### TMA cases and construction

The TMA cohort consisted of cases selected randomly by the study statistician (ZF) according to de-identified clinical data from each site such that recurrent and non-recurrent cases were balanced. Constraints were placed on case selection such that patients with recurrence and with Gleason score 3+3 = 6 and those with Gleason score 4+4 = 8 and no recurrence were oversampled. Details concerning case selection, tissue microarray construction and statistical considerations have been described elsewhere [20]. TMAs were constructed at each participating center using 1 mm cores and a standardized TMA layout. For each case, 3 cores of the highest grade cancer from the largest cancer area were used as well as one core of histologically normal prostate tissue from each case. In each TMA block at all sites, a common set of tissue cores (colon, tonsil, kidney, healthy prostate, and liver) was included as a staining control and for normalization across TMAs. Thereafter, the TMAs were baked and stored under nitrogen gas at each site.

### Immunohistochemistry

Immunohistochemical staining was performed using freshly cut 5 micron sections from each site shipped to Stanford University and a commercial antibody for MUC1 (1:50 dilution; SC-7313, Santa Cruz Biotechnology) [20]. The digital image documentation of all stained slides was performed using the Leica SCN400 scanning system with the SL801 autoloader (Leica Microsystems; Concord, Ontario, Canada) at magnification equivalent to 40x. The images were transferred into the SlidePath digital imaging hub (DIH; Leica Microsystems). In parallel, separate TMA sections were stained with hematoxylin and eosin (H & E) and high molecular weight keratins (HMWK, 34bE12, Dako); these sections were scored for the presence of cancer in each core on the TMA as described previously [21–26]. A single pathologist (LF) scored MUC1 protein staining only in cores in which cancer was present as determined using the H & E and HMWK.

**Fig 1:**
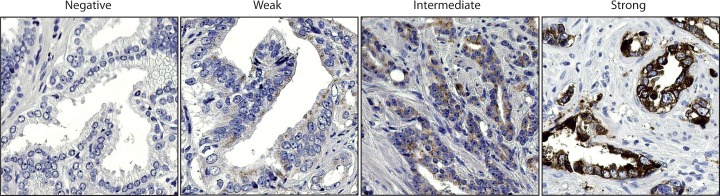
Immunohistochemical MUC1 staining in representative prostate cancer samples showing absent, weak, moderate and strong staining.

The immunohistochemical staining intensity for MUC1 was defined as absent, weak (faint cytoplasmic staining of scattered cells), moderate (intermediate or heterogeneous cytoplasmic staining in tumor cells), or strong (dense cytoplasmic staining of nearly all tumor cells) as shown in **Fig 1**.

### Statistical methods

The clinical and pathological characteristics were comprised of age, pre-surgery PSA, post-surgical Gleason score, seminal vesicle invasion (SVI), extra-capsular invasion (ECE), and surgical margin status. Patient characteristics (e.g. race, lymph node status, etc.) with 25% or more missing were excluded from this analysis. Subjects with evaluable MUC1 staining, clinical and pathological data were included in the analysis.

The outcomes of interest included post-surgical recurrence-free survival (RFS), Disease-specific survival (DSS) and overall survival (OS). RFS was defined as the absence of PSA (biochemical) recurrence, local recurrence, CaP metastases, or death from CaP, with events determined at the earliest date noted after surgery. The endpoint of DSS was defined as death from CaP or development of metastatic disease. The endpoint of OS was defined as death from any cause. The date of surgery was considered as baseline for survival analysis. MUC1 IHC score was the maximum staining score of all cores for each patient. As described previously, MUC1 stained cases were divided into two groups, “negative” vs. “positive” (weak/moderate/strong staining), and compared to each other [5].

Descriptive statistics of patients’ MUC1 protein expression by IHC were recorded as frequencies and percentages for the patient cohort. The association between MUC1 expression levels and categorical values was assessed by the Chi-square test. The Wilcoxon rank sum test was performed to evaluate the association between MUC1 expression and continuous variables. The Kaplan-Meier (KM) method was used to determine RFS, DSS and OS by MUC1 expression groups. We used the log-rank test to find significant differences between survival curves. Univariable and multivariable Cox regression analyses were performed to evaluate the prediction of MUC1 expression for each survival endpoint. Unweighted and weighted analyses were performed, with the latter accounting for the oversampling of patients with recurrence less than 5 years after surgery. All of the statistical tests were 2-sided, and the level of statistical significance was P < 0.05. Statistical analysis was performed using SAS version 9 (SAS Institute, Cary, NC). Kaplan Meier plots were created using Spotfire S+ 8.2 (TIBCO Inc., Palo Alto, CA). The complete dataset of clinical, pathological and staining data can be found in S1 File.

## Results

The TMA was constructed from radical prostatectomy specimens from a total of 1,326 subjects. Of those cases, >25% of clinical or pathological data were missing in 51 cases (3.8%). MUC1 staining data were not available in 95 cases (7.2%) due to core loss or lack of cancer in the core samples. After excluding those cases, the remaining 1,180 cases with available clinical, pathological and IHC data constitute the cohort of the current study. Overall, 73.3% (865/1,180) showed absent MUC1 expression, 11.9% (140/1,180) showed weak expression, 9.2% (109/1,180) showed moderate expression, and 5.6% (66/1,180) showed strong expression. When MUC1 expression status was divided into “positive” and “negative” status, 26.7% of cases were scored with positive expression, whereas 73.3% of cases were negative.

### MUC1 and clinicopathological features

MUC1 levels by IHC were tested for their association with clinical and pathologic features (**Table 1**). Initially we tested degree of staining (absent, weak, moderate, strong) for association with pre-operative clinical and pathological data and found no association of degree of staining and the presence of ECE, SVI, positive surgical margins, Gleason score and pre-operative PSA. However, patients showing a negative or weak status for MUC1 expression were younger than those with moderate or strong status. Since our goal was to validate whether MUC1 staining is a prognostic biomarker in CaP tissues, we simplified MUC1 staining into any positive staining (weak, moderate or strong) compared to absent staining since this was how MUC1 was scored in previous positive studies [5, 19, 27]. The presence of any MUC1 staining was associated with extracapsular extension (ECE) and higher Gleason score (GS) (**Table 1**). No significant association was observed between MUC1 expression and seminal vesicle invasion (SVI), patient age at the time of surgery, positive surgical margins (PSM) or pre-operative serum PSA levels. Lymph node status was missing for approximately half of the cases and therefore was not included in our analysis.

**Table 1:** MUC1 expression and clinical and pathological features.

	All	MUC1 Score		P-value
		Negative	Positive	
Population, n (%)	1180 (100%)	315 (26.7%)	865 (73.3%)	
Age at diagnosis, median (range), yr.	61 (35–80)	61 (35–78)	62 (42–80)	0.13*
Preoperative PSA level, mean (+/-SD), ng/mL	8.63+/-8.36	8.71+/-8.60	8.55+/-8.12	0.78*
Surgical margin status				0.19**
Positive, n (%)	347 (29.41)	259 (74.64)	88 (25.36)	
Negative, n (%)	666 (56.44)	471 (70.72)	195 (29.28)	
Unknown, n (%)	167 (14.15)	135 (80.84)	32 (19.16)	
Seminal vesicle invasion				0.57**
Yes, n (%)	78 (6.61)	55 (70.51)	23 (29.49)	
No, n (%)	1086 (92.03)	798 (73.48)	288 (26.52)	
Unknown, n (%)	16 (1.36)	12 (75.00)	4 (25.00)	
Extracapsular Extension				0.02**
Yes, n (%)	347 (29.41)	238 (68.59)	109 (31.41)	
No, n (%)	818 (69.32)	617 (75.43)	201 (24.57)	
Unknown, n (%)	15 (1.27)	10 (66.67)	5 (33.33)	
Gleason score				0.02**
< = 6, n (%)	494 (41.86)	382 (77.33)	112 (22.67)	
7a (3+4), n (%)	436 (36.95)	315 (72.25)	121 (27.75)	
7b (4+3), n (%)	135 (11.44)	93 (68.89)	42 (31.11)	
8–10, n (%)	107 (9.07)	69 (64.49)	38 (35.51)	
Unknown	8 (0.68)	6 (75.00)	2 (25.00)	

* Wilcoxon rank sum test

** Chi-square test

### MUC1 and clinical outcomes after radical prostatectomy

**Fig 2:**
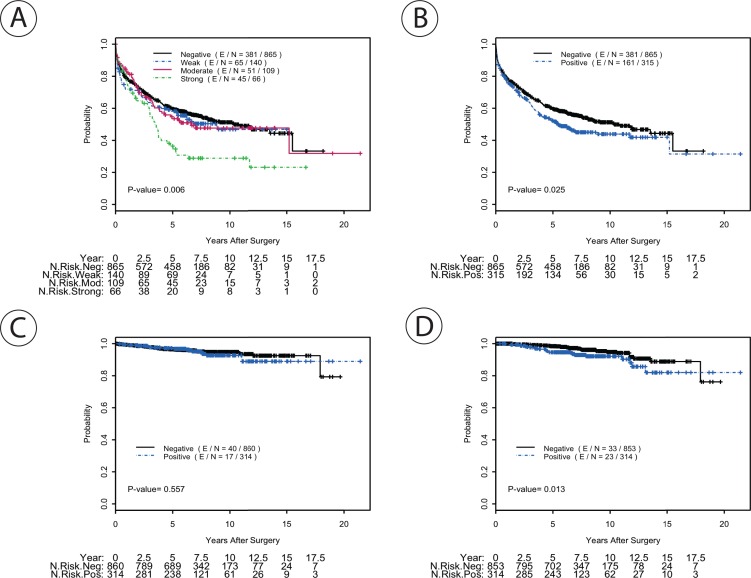
Kaplan-Meier plots of recurrence free survival (RFS) after radical prostatectomy **A)** for MUC1 staining gradient (absent, weak, moderate, and strong staining); **B)** for categorized MUC1 staining status (negative vs. positive); **C)** disease-specific survival for MUC1 positive and negative staining; **D)** Overall survival for the MUC1 positive and negative staining cases.

Kaplan-Meier analysis showed that the strong MUC1 expression was significantly associated with worse RFS compared to negative, weak, or moderate MUC1 expression as shown in **Fig 2A** (P = 0.006, Log-rank test). When the cohort was stratified as either positive (weak, moderate, strong) or negative MUC1 staining, cases that were positive for MUC1 showed relatively slight but significantly worse RFS compared to those that were negative (**Fig 2B**). MUC1 expression was not associated with DSS when cases were grouped by their degree of staining (negative, weak, moderate, strong; not shown) or simply divided into positive or negative staining (**Fig 2C**). Patients with positive MUC1 staining had a slightly worse OS compared to those without (P = 0.013, Log rank test), although there was no significant difference in outcome when each staining group was considered individually (Not shown, P = 0.16, Log-rank test) (**Fig 2D**).

To further explore the relationship between MUC1 expression levels and clinical outcomes, we performed univariable Cox proportional hazards analysis for MUC1 expression (positive or negative), as well as clinical and pathological variables (**Table 2**). Patients with positive MUC1 staining had significantly a worse RFS (HR: 1.23, P = 0.02). RFS was also strongly associated with the presence of ECE, SVI, PSM, increasing pre-operative PSA and increasing GS, but not with patient age. DSS was associated with all of the clinical variables, but not with MUC1 staining status or patient age. OS was associated strongly with the presence of high GS, age, and to a lesser extent with SVI, ECE, Pre-operative PSA and MUC1 staining (P = 0.02). Analysis of MUC1 expression degree by staining (negative, weak, moderate strong) slightly strengthened the association with RFS (P = 0.007), but did not change the association with DSS (P = 0.24) or OS (P = 0.06).

**Table 2:** Univariate Cox proportional hazard model for recurrence-free survival, disease-specific survival and overall survival.

	Recurrence-free survival		Disease-specific survival		Overall survival	
	HR (95% CI)	P-value	HR (95% CI)	P-value	HR (95% CI)	P-value
Age	1.00 (0.99–1.01)	0.65	1.02 (0.99–1.06)	0.22	1.08 (1.03–1.12)	0.0004
Log(preoperative PSA)	2.17 (1.54–3.07)	<0.0001	2.17 (1.54–3.07)	<0.0001	1.68 (1.12–2.52)	0.01
MUC1						
Negative	Reference		Reference		Reference	
Positive	1.23 (1.03–1.49)	0.02	1.19 (0.67–2.08)	0.56	1.92 (1.14–3.33)	0.02
Surgical margin status						
Negative	Reference		Reference		Reference	
Positive	2.08 (1.74–2.48)	<0.0001	2.65 (1.43–4.91)	0.002	1.61 (0.95–2.72)	0.08
Seminal Vesicle Invasion						
No	Reference		Reference		Reference	
Yes	3.33 (2.63–4.35)	<0.0001	3.45 (1.82–6.67)	0.002	2.5 (1.18–5.26)	0.02
Extracapsular extension						
No	Reference		Reference		Reference	
Yes	1.92 (1.61–2.27)	<0.0001	1.96 (1.16–3.33)	0.01	1.69 (0.99–2.86)	0.05
Gleason score						
< = 6	Reference		Reference		Reference	
3+4 (7a)	1.43 (1.18–1.74)	0.0003	2.93 (1.43–6.00)	0.003	0.95 (0.48–1.88)	0.88
4+3 (7b)	2.39 (1.87–3.06)	<0.0001	3.71 (1.53–8.99)	0.004	1.42 (0.57–3.53)	0.45
8–10	2.39 (1.82–3.13)	<0.0001	7.30 (3.30–16.12)	<0.001	4.79 (2.52–9.11)	<0.0001

To evaluate whether MUC1 expression levels provided prognostic information independent of clinical variables, we performed multivariable Cox proportional hazards analysis (**Table 3**). MUC1 expression levels (positive/negative or absent/weak/moderate/strong) were not associated with RFS or DSS. As reported previously [21–26], RFS was associated with the presence of ECE, PSM, SVI, increasing Gleason score and higher pre-operative PSA. DSS in this cohort was only associated with Gleason score and pre-operative PSA levels. For OS, MUC1 did show a significant association (HR 1.82; 95% CI: 1.06–3.11; P = 0.03) as did GS and patient age. However, the associations between MUC1 staining and DSS and OS were limited by the relatively small number of CaP deaths or metastasis (n = 57) or deaths from all causes (n = 56).

**Table 3:** The multivariate cox proportional hazard model for recurrence-free survival.

	Recurrence-free survival		Overall survival	
	HR (95% CI)	P-value	HR (95% CI)	P-value
Age			1.07 (1.02–1.11)	0.003
Log(preoperative PSA)	1.42 (1.22–1.67)	<0.0001		
MUC1				
Negative	Reference		Reference	
Positive	1.14 (0.92–1.42)	0.23	1.82 (1.06–3.11)	0.03
Surgical margin status				
Negative	Reference			
Positive	1.64 (1.32–2.03)	<0.0001		
Seminal Vesicle Invasion				
No	Reference			
Yes	2.10 (1.52–2.90)	<0.0001		
Extracapsular extension				
No	Reference			
Yes	1.30 (1.04–1.62)	0.02		
Gleason score		0.0001		0.0005
< = 6	Reference		Reference	
3+4 (7a)	1.20 (0.94–1.53)		0.89 (0.45–1.77)	
4+3 (7b)	1.92 (1.43–2.58)		1.17 (0.47–2.95)	
8–10	1.50 (1.07–2.09)		3.46 (1.76–6.78)	

## Discussion

In a large multi-institutional clinical cohort, we have demonstrated that expression of MUC1 protein by immunohistochemistry is associated with extracapsular extension and high Gleason grade at the time of radical prostatectomy. This association confirms several smaller studies that have noted an association of MUC1 protein expression and increasing Gleason grade [8, 18, 28, 29], and disagrees with another study (N = 110) that showed no association of MUC1 expression with pathological features [30]. The association of MUC1 expression with adverse pathological features suggests that MUC1 could have utility as a biomarker for predicting tumor upgrading or upstaging. Because of sampling errors in biopsies, approximately 40% of Gleason score 3+3 = 6 cancers on pre-operative biopsy are found to be ≥ 7 at the time of radical prostatectomy [31]. Under grading and under staging are significant challenges when selecting men with apparent low risk CaP for active surveillance, and likely account for significant rates of adverse reclassification for men while on surveillance [32]. The potential for MUC1 to predict adverse reclassification has been suggested by a demonstration that MUC1 expression independently predicted upstaging and upgrading in low risk prostate cancers incidentally discovered at the time of transurethral resection of the prostate. These cases were treated for benign prostatic hyperplasia and subsequently underwent radical prostatectomy [33].

Despite its association with adverse pathological features, MUC1 expression did not predict outcome independent of Gleason score, extracapsular extension, seminal vesicle invasion, positive surgical margins and pre-operative PSA levels. Previous reports have implicated MUC1 as a potential prognostic biomarker prostate cancer. Lapointe et al. showed that MUC1 expression was independently associated with RFS in a cohort of 225 patients after surgery, although in this study, Gleason score and stage were dichotomized as ≤3+4 compared to ≥4+3 and ≤pT2 vs. ≥pT3, respectively [5]. In a population-based study of 195 Swedish men managed by watchful waiting, MUC1 expression that deviated from normal was independently associated with disease specific survival [17]. However, deviation from normal was defined as staining above and below levels in normal prostate tissue, and cases with absent expression showed outcomes similar to those with high expression, a finding that differs from our findings and is difficult to explain biologically.

One significant challenge in developing MUC1 as a prognostic biomarker is that the protein is heavily glycosylated, and the glycoforms change in CaP compared to normal prostate tissue. In prostate cancer, as in many malignancies, MUC1 and other glycoproteins show truncated O-glycans and an increase in sialylation [18, 34]. The changes in glycosylation are driven in part by increased expression of the glycoprotein synthetic enzyme GCNT1 (β-1,6-N-acetylglucosaminyltransferase-1) in CaP compared to normal prostate tissues, which is associated with an increase in sialylated MUC1 [6]. Using an antibody specific for sialylated MUC1, Arai et al. found high level expression by IHC was associated with higher grade and stage of prostate cancer as well as RFS and DSS [18]. However, the alterations in glycosylation patterns in cancer, as well as potential heterogeneity in the glycosylation patterns in cancer could complicate analyses of MUC1 expression in tissues and degrade its performance as a biomarker. For example, using a panel of antibodies specific to different glycoforms of MUC1, Burke et al. found significant differences in MUC1 expression and this dramatically affected the associations between MUC1 over-expression and pathological outcomes. Only the antibodies directed at less glycosylated forms of MUC1 demonstrated an association with adverse pathology [35]. The variation in staining results between the specific antibodies implies that there could be some heterogeneity in glycosylation patterns that could adversely affect the performance of MUC1 as a biomarker.

The finding of increased MUC1 expression in cancers with adverse pathologic features suggests that MUC1 could play a role in prostate cancer progression. MUC1 has been implicated in cancer progression in many model systems and has been shown to modulate cancer cell adhesion and migration, evasion of immune surveillance, and cancer cell signaling [34, 36]. In CaP, MUC1 expression is significantly higher in synchronous lymph node metastases compared to primary tumors and is correlated with adverse outcome [29, 37]. MUC1 expression has also been reported in prostate cancer metastatic to the bone [38]. Therefore, MUC1 might have an important role in prostate cancer progression, and has been considered as a potential therapeutic target in advanced disease [39].

Our study has some limitations. Patient samples were collected retrospectively and, although we tried to limit biases by using a case control design, potential confounders are possible including changes in practice patterns or patient populations over time. Rather than select cases that reflect the distribution of GS and RFS typical of the population of patients undergoing radical prostatectomy, we over-sampled recurrent low grade (GS 3+3 = 6), balanced recurrent and non-recurrent cases with GS 3+4 = 7 and 4+3 = 7 and oversampled non- recurrent GS≥8 cancers. While this design has advantages in identifying biomarkers independent of GS, it will diminish the weight of GS in univariate and multivariate models in predictions of clinical outcome.

In summary, MUC1 expression is associated with extracapsular extension and higher Gleason score in men undergoing radical prostatectomy for clinically localized prostate cancer. However, MUC1 expression is not a prognostic biomarker since it is not an independent predictor of clinical outcome following surgery. Given its association with adverse pathology, MUC1 could have some role in selecting patients for definitive treatment who otherwise have features of low risk prostate cancer.

## Supporting Information

S1 File Raw clinical, pathological and staining data from the cohort. (XLS)
